# Left ventricular function and mechanics following prolonged endurance exercise: an update and meta-analysis with insights from novel techniques

**DOI:** 10.1007/s00421-018-3906-z

**Published:** 2018-06-05

**Authors:** Rachel N. Lord, Victor Utomi, David L. Oxborough, Bryony A. Curry, Megan Brown, Keith P. George

**Affiliations:** 1 Cardiff Centre for Exercise and Health, Cardiff Metropolitan University Cyncoed Campus, Cyncoed Road, Cardiff, CF236XD UK; 2 Research Institure for Sport and Exercise Sciences, Tom Reilly Building, Liverpool John Moores University, Liverpool, UK

**Keywords:** Left ventricular mechanics, Endurance exercise, Echocardiography

## Abstract

**Background:**

The cardiac consequences of undertaking endurance exercise are the topic of recent debate. The purpose of this review is to provide an update on a growing body of literature, focusing on left ventricular (LV) function following prolonged endurance exercise over 2 h in duration which have employed novel techniques, including myocardial speckle tracking, to provide a more comprehensive global and regional assessment of LV mechanics.

**Methods:**

Prospective studies were filtered independently following a pre-set criteria, resulting in the inclusion of 27 studies in the analyses. A random-effects meta-analysis was used to determine the weighted mean difference and 95% confidence intervals (CI) of LV functional and mechanical data from pre-to-post-exercise. Narrative commentary was also provided where volume of available evidence precluded meta-analysis.

**Results:**

A significant overall reduction in LV longitudinal strain (*Ɛ*) *n* = 22 (− 18 ± 1 to − 17 ± 1%; effect size (*d*) − 9: − 1 to − 0.5%), strain rate *n* = 10 (SR;*d* − 0.9: − 0.1.3 to − 0.5 l/s) and twist*n* = 5 (11.9 ± 2.2 to 8.7 ± 2.2°,*d* − 1: − 1.6 to − 0.3°) was observed following strenuous endurance exercise (range 120–1740 min) (*P* < 0.01). A smaller number of studies (*n* = 4) also reported a non-significant reduction in global circumferential and radial *Ɛ* (*P* > 0.05).

**Conclusion:**

The meta-analysis and narrative commentary demonstrated that a reduction in LV function and mechanics is evident following prolonged endurance exercise. The mechanism(s) responsible for these changes are complex and likely multi-factorial in nature and may be linked to right and left ventricular interaction.

## Introduction

Prolonged strenuous exercise is growing in popularity (Hoffman 2016). Increased participation has led to a growth in related research including the controversial topic of a transient reduction in cardiac function post-exercise (Middleton et al. 2006). This has been termed “exercise induced cardiac fatigue” (EICF) (Douglas et al. 1987). Originally proposed as a concept by Saltin and Stenberg (1964), this phenomenon has received more attention as cardiac imaging tools have improved (Dawson et al. 2008; Shave et al. 2009; Oxborough et al. 2010a, b). The prevalence, causes and consequences of altered cardiac function after prolonged strenuous exercise have prompted on-going empirical study and debate (Douglas et al. 1987; Lord et al. 2016) that has led to a number of narrative reviews (Dawson et al. 2005; Shave et al. 2008; Oxborough et al. 2010a, b) and meta-analyses (Middleton et al. 2006; Elliott and La Gerche 2015). In a global context, during recovery from prolonged endurance exercise, heart rate and therefore cardiac output are elevated, however despite this, there is evidence to suggest that there is an intrinsic reduction in cardiac function.

Studies completed between the 1980s and 2006 were compiled within a meta-analysis undertaken by Middleton et al. (2006). The outcome variables were ejection fraction (EF), a gross estimate of left ventricular (LV) contractile function, and the early to atrial (*E*/*A*) peak transmitral flow velocity ratio, an index of global diastolic function. In a collapsed sample of 294 athletes, completing between 1 and 24 h of prolonged exercise, there was a significant overall effect on both EF (2% decline) and*E*/*A* (0.5*au* decline) post-exercise. The outcome for EF was partially mediated by preload, exercise duration and training level although this was not the case for *E*/*A*. Study-to-study heterogeneity, possibly linked to exercise mode, training status of participants, technical measurement issues and study design limitations have been documented in later narrative reviews (Shave et al. 2008; Oxborough et al. 2010a, b). Whilst empirical data continues to be collected (Neilan et al. 2006a, b; Hart et al. 2007; Dawson et al. 2008; La Gerche et al. 2008, 2011, 2012, 2015; George et al. 2009; Nottin et al. 2009; Sahlen et al. 2009; Scott et al. 2009; Shave et al. 2009; Banks et al. 2010, 2011; Chan-Dewar et al. 2010; Oxborough et al. 2010b, 2011; Oosthuyse et al. 2012; Vitiello et al. 2013a, b; Dalla Vecchia et al. 2014; Cote et al. 2015; Stewart et al. 2015; Lord et al. 2016), the most recent meta-analysis (Elliott and La Gerche 2015) focussed solely on the right ventricular response to prolonged exercise. Consequently, this new review and meta-analysis will update our understanding of the LV response to prolonged exercise.

A new systematic review and meta-analysis is timely because of; (1) the continuing interest and development of a growing and often contradictory or underpowered empirical database, and (2) the substantial developments in cardiac imaging technology that are providing a more “complete” picture of cardiac function, motion and mechanics in different planes of motion as well as in specific regions of the myocardium (Oxborough et al. 2006; George et al. 2009). Tissue Doppler imaging (TDI) was adopted in EICF research in an attempt to overcome some of the load-dependent limitations of standard 2D and Doppler echocardiographic techniques as well as providing local or regional functional assessment (George et al. 2005). Despite this tissue Doppler is influenced by translation, tethering and the angle of insonation (Marwick 2006). The advent of myocardial strain (*ε*) imaging can overcome these issues and tissue Doppler *ε* imaging allowed the assessment of LV Eulerian *ε* and strain rate (SR) providing regional and global assessment of cardiac function (Neilan et al. 2006a, b). Because tissue Doppler *ε* remains angle-dependent and cardiac mechanics occur in mutliple planes of motion the majority of *ε* and SR data acquired in EICF research has employed myocardial speckle tracking technology to determine regional and global Lagrangian *ε* and SR data in multiple planes. This imaging tool also facilitates the estimation of LV rotation, twist and untwist. LV untwisting is fundamental in the development of an intra-ventricular pressure gradient that drives early diastolic filling (Notomi et al. 2008) and thus provides further insight within the post-exercise setting.

There have been no systematic or narrative reviews of the LV responses to prolonged strenuous exercise since 2010 (Oxborough et al. 2010a). An up-to-date review and meta-analysis including new studies employing tissue Doppler and ε imaging techniques provides a timely update on our knowledge as well as drive on-going discussions about the potential physiological mechanism(s) underpinning EICF. Potential physiological mechanisms and new data on ventricular interaction will be reviewed after the presentation and discussion of data from both meta-analysis outcomes and narrative comment.

## Methods

Our initial aim was to identify all echocardiographic studies examining tissue Doppler and myocardial *ε* parameters following a bout of endurance exercise > 120 min in duration. Relevant MeSH subject terms and keywords pertaining to post-endurance exercise cardiac functional response and Boolean operators were used in online database searches. In addition to date limits (2006 onwards), the search was limited to human studies and those with an English language abstract. The following search string was employed:

- ‘Left Ventricular Strain$ OR Mechanics$ AND Endurance$ Exercise$’

**Fig. 1:**
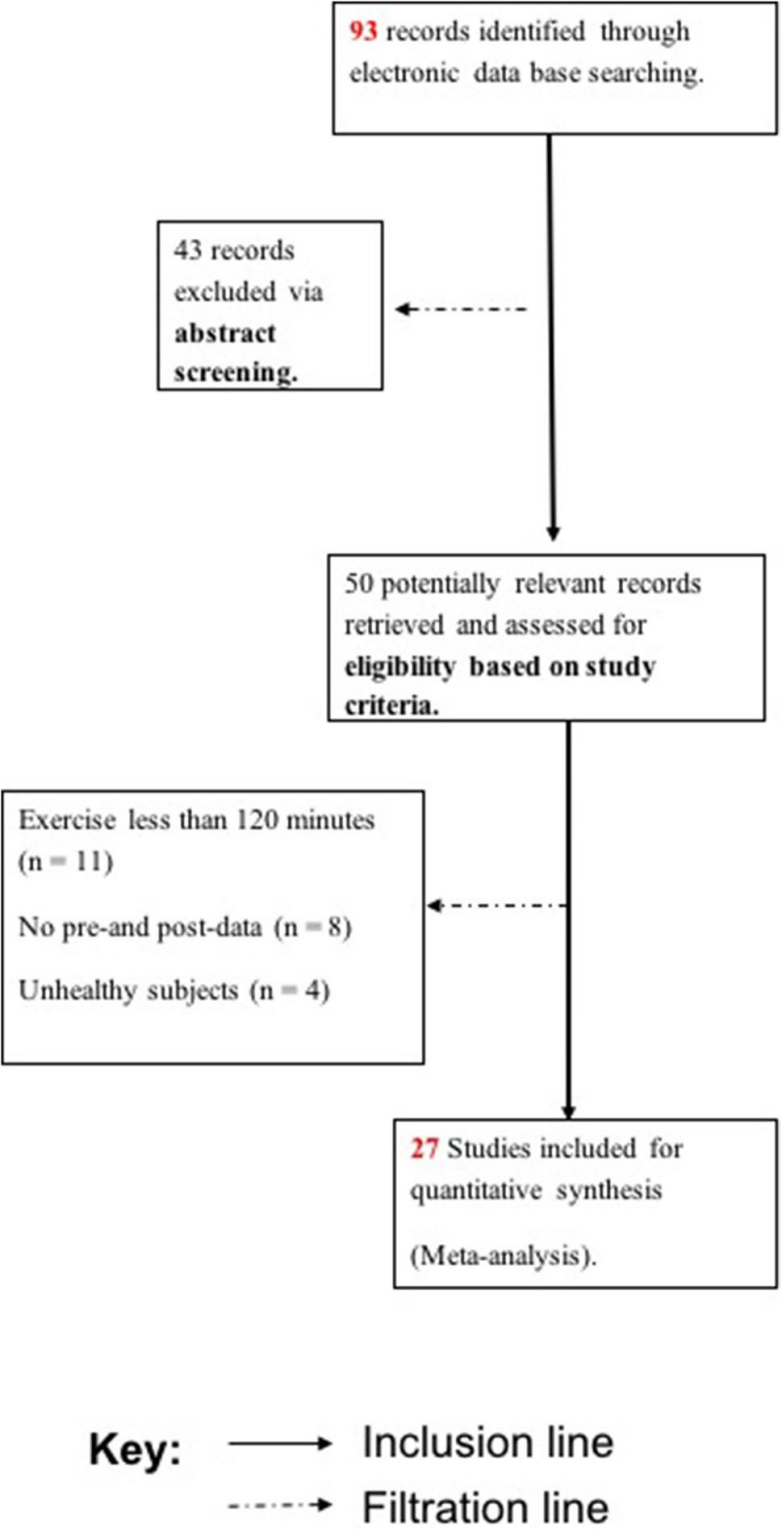
Outline of the search and filtration process for studies included in the meta-analysis

Prospective studies were filtered initially using titles and then abstracts. This process was completed independently by two authors (MB, BC) who compared decision-making and discussed disagreements. Inclusion criteria were: (1) Exercise duration over 120 min, (2) Pre-and post-exercise data provided and (3) Healthy subjects with no history of cardiovascular disease. This resulted in 27 studies for inclusion in the meta-analysis (Fig. 1; Table 1).

**Table 1:** Study and participant demographics, training history and exercise stimulus detail

Study	*N*	Male	Female	Age (years)	Distance (km)	Duration (min)	Exercise type	Training distance (km)	Training time (min)
Banks et al. (2011)	18	12	6	28		150	High-intensity exercise		
Banks et al. (2011)	18	15	3	52		150	High-intensity exercise		
Chan Dewar et al. (2010)	19	16	3	41	89	586	Ultramarathon	88	
Cote et al. (2015)	17	17	0	45	160	1740	Run	86	
Cote et al. (2015)	8	0	8	46	160	1554	Run	86	
Dalla Vecchia et al. (2014)	35	31	4	42	160		Half marathon		
Dawson et al. (2008)	15	15	0	32	42	229	Marathon		
George et al. (2009)	19	16	3	41	89	586	Run		
Hart et al. (2007)	14	13	1	34	42	126	Marathon		
La Gerche et al. (2008)	27	20	7	32		600	Triathlon		1152
La Gerche et al. (2011)	39	35	4	36					960
La Gerche et al. (2011)	14	12	2	38					102
La Gerche et al. (2012)	40	36	4	37			Run/tri/cycle/ultra		
La Gerche et al. (2015)	40	36	4	37			Marathon, endurance triathlon, alpine cycling race and an ultra-triathlon		978
Lord et al. 2016	15	14	1	40	160		Ultramarathon	104	
Neilan et al. (2006a, b)	60			41	42	245	Run	67.2	
Nottin et al. (2009)	23	23	0	40		840	Triathlon		720
Oosthuyse et al. (2012)	11	11	0	30			Stimulated race cycling		780
Oxborough et al. (2010a, b)	17	17	0	33	42	209	Marathon		
Oxborough et al. (2011)	16	12	4	42	161	1470	Run	104	
Sahlen et al. (2009)	15	15	0	62	30	199	Cross-country race		276
Scott et al. (2009)	25	20	5	41	160	1530	Ultramarathon		786
Shave et al. (2009)	15	14	1	32	42	213	Marathon		
Stewart et al. (2015)	10	10	0	27		120			480
Vitiello et al. (2013a, b)	21	21	0	40	166	2280	Ultramarathon		
Vitiello et al. (2013b)	16	16	0	23		180	Cycling (ergometer)		

All relevant cardiac data were extracted (MB, BC) directly from individual trials into a spreadsheet (Excel 2010, Microsoft Corp). Continuous data for LV functional parameters were recorded as group mean ± SD for each study. Extracted systolic variables comprised ejection fraction (EF), systolic septal mitral annular tissue velocity (*S*′), longitudinal, radial and circumferential ε and systolic SR (SRS), rotation and systolic rotation rates as well as twist. Diastolic variables comprised early diastolic transmitral blood flow velocity (*E*), late diastolic transmitral blood flow velocity (*A*), early diastolic mitral annular tissue velocity (*E*′), late diastolic mitral annular tissue velocity (*A*′), longitudinal, radial and circumferential diastolic SR (ESR and ASR) as well as untwist. Outcome variables selection was based upon physiological relevance and study-to-study reporting. Furthermore, we extracted data on heart rate, blood pressure and LV end diastolic volume (LVEDV) as potential mediating factors (rate, afterload, preload) for LV function.

### Statistical analysis

**Fig. 2:**
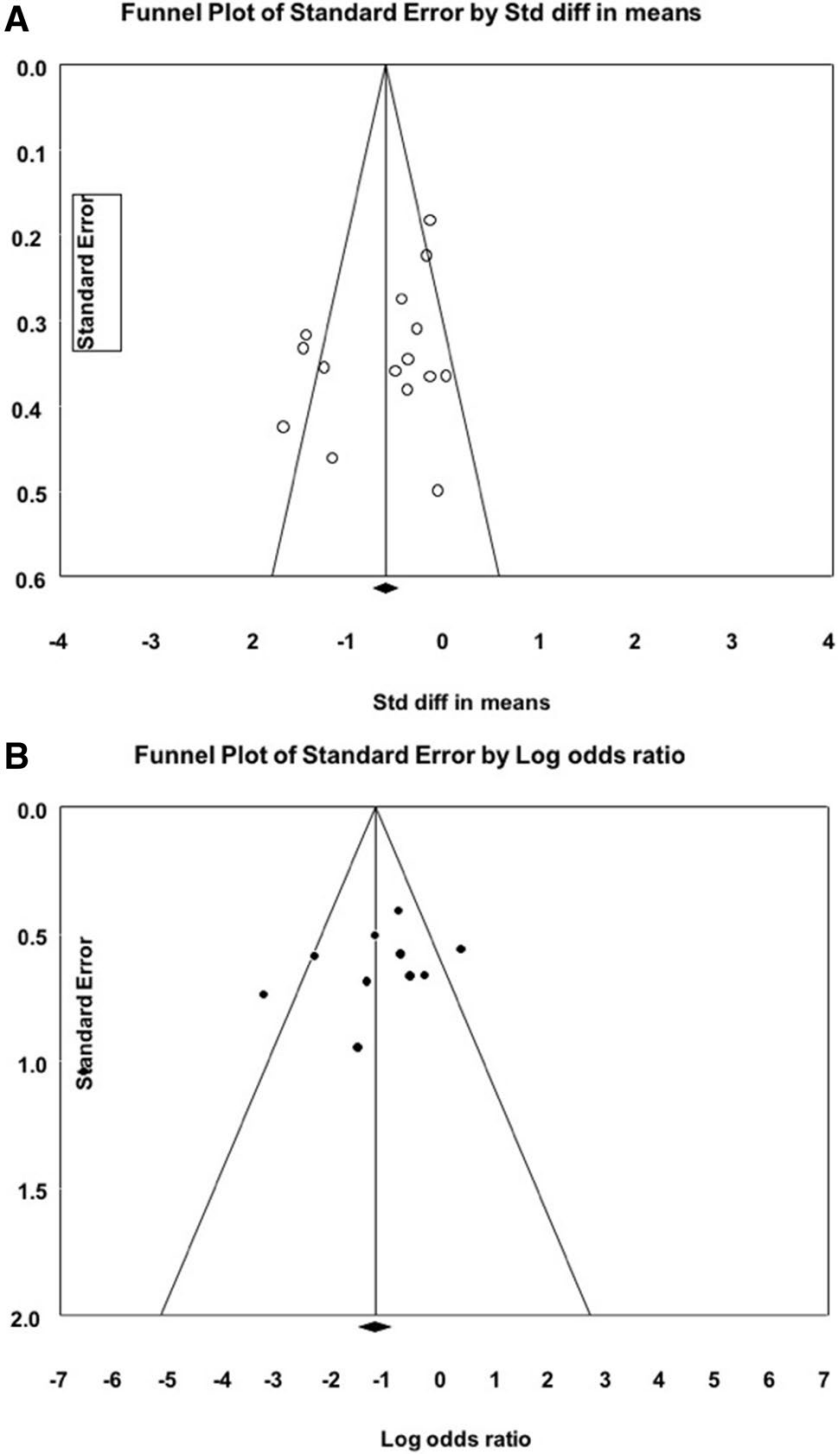
Exemplar funnel plots for (**A**) Ejection fraction and (**B**) longitudinal strain

A random-effects meta-analysis was used to determine the weighted mean difference (WMD) and 95% CIs of LV functional data between pre-exercise and post-exercise as well as data for HR, blood pressure and LVEDV. For the purposes of the meta-analytic technique we focused on specific primary LV functional variables on the basis of the number of studies containing relevant data. Where data were available for ≥ 4 studies a meta-analysis approach was completed. For variables with fewer studies narrative comparisons and comments were made. All statistical analyses were performed using Comprehensive Meta-Analysis (Biostat: V 2.2.064, Englewood, NJ, USA). For comparison of moderator variables, standardised difference in means (Cohen’s d)/effect sizes were calculated for each individual study and a summary with overall effect size recorded for each group of studies. Negative effect sizes indicated greater moderator variable at pre-exercise group, whereas a positive effect size identified greater value post-exercise. Heterogeneity was reported using Cochran’s Q and *I*^*2*^ statistic (the percentage of total variation between studies due to heterogeneity rather than chance). To address publication bias, funnel plots were calculated following Egger’s regression intercept. Exemplar plots for EF and longitudinal ε are provided in Fig. 2a, b. The funnel plots demonstrated that fewer studies were outside of the funnel but were distributed to the right. This supports further evaluation of study-to-study heterogeneity.

## Results and commentary

### Loading conditions

Following prolonged endurance exercise, heart rate (HR) was significantly elevated from 59 ± 1 (56–61) to 78 ± 2 (76–81) whilst SBP was significantly reduced from 126 ± 2 (121–131) to 115 ± 3 (109–132). LV EDV was significantly reduced from 125 ± 11 to 117 ± 11 ml pre-to-post-exercise [*d* = − 0.53, 95% CI (− 0.8 to − 0.3, *P* < 0.001)]. LV systolic and diastolic functional parameters should be interpreted with consideration of these changes in loading conditions coupled with the significant evidence of study-to-study heterogeneity (*I*^2^ statistic > 75% for most variables; see Table 2). Although both an increase in inotropic stimulation and a decrease in afterload are associated with an increase in systolic and diastolic functional parameters, a reduction in preload may have an independent and negative impact on LV systolic and diastolic function.

**Table 2:** Meta-analysis of LV functional parameters pre- and post-exercise endurance exercise (> 120 min)

Parameter	Number of studies	Pre mean	Post mean	*P* value	*d*	95% CI	Heterogeneity		
							Cochrane’s *Q*	*I*^2^ statistic (%)	*P* value
Systolic function									
HR (bpm)	20	59 ± 1 (56 to 61)	78 ± 2 (76 to 81)	0.01	2	2–4	52	67	0.01
SBP (mmHg)	16	126 ± 2 (121 to 131)	115 ± 3 (109 to 132)	0.01	− 1	− 1.5 to − 0.6	30	73	0.01
DBP (mmHg)	11	74 ± 1 (72 to 76)	71 ± 2 (68 to 75)	0.01	− 0.5	− 0.8 to 0.3	4	0	0.5
LVEDV	13	125 ± 11 (104 to 147)	117 ± 11 (95 to 138)	0.01	− 0.5	− 0.8 to − 0.3	17	28	0.2
EF (%)	17	63 ± 1.0 (62 to 65)	60 ± 1.0 (58 to 62)	0.01	− 0.8	− 1.2 to − 0.5	74	78	0.01
*S*′ (cm/s)	5	9.5 ± 0.8 (7.8 to 1.1)	9.7 ± 0.9 (7.9 to 11.4)	0.08	− 0.7	− 1 to − 0.4	2	0	0.2
Longitudinal strain (%)	22	− 18 ± 1 (− 19 to − 17)	− 17 ± 1 (− 18 to − 16)	0.01	− 0.9	− 1 to − 0.5	44	77	0.1
Long SSR (l s^−1^)	10	− 0.99 ± 0.03 (− 1.04 to − 0.90)	− 1.01 ± 0.03 (− 1.07 to − 0.96)	0.01	− 0.9	− 1.3 to − 0.5	44	77	0.01
Circumferential strain (%)	4	− 19.2 ± 1.1 (− 21.4 to − 17.0)	− 17.8 ± 1.1 (− 19.9 to − 15.7)	0.2	− 1.1	− 2.8 to 0.5	42	93	0.01
Radial strain (%)	4	27.4 ± 13.0 (1.8 to 53.0)	11.2 ± 16.8 (− 21.8 to 44.2)	0.07	− 1	− 2.5 to 0.1	28	89	0.04
Twist (^o^)	5	11.9 ± 2.2 (7.7 to 16.2)	8.7 ± 2.2 (4.5 to 13.0)	0.01	− 1	− 1.6 to − 0.3	19	78	0.01
Diastolic function									
*E* (m/s)	9	0.8 ± 0.02 (0.7 to 0.8)	0.7 ± 0.02 (0.6 to 0.7)	0.01	− 1	− 1.4 to − 0.6	50	74	0.01
*A* (m/s)	14	0.5 ± 0.02 (0.5 to 0.5)	0.6 ± 0.02 (0.5 to 0.6)	0.01	− 0.6	− 0.9 to − 0.2	49	73	0.01
*E*/*A*	18	1.6 ± 0.1 (1.5 to 1.7)	1.3 ± 0.1 (1.2 to 1.4)	0.01	− 1.1	− 1. to − 0.8	59	71	0.01
*E*′ (cm/s)	14	10 ± 1 (9 to 11)	9 ± 1 (8 to 11)	0.01	− 0.7	− 1 to − 0.5	15	39	0.1

*HR* heart rate, *SBP* systolic blood pressure, *DBP* diastolic blood pressure, *EF* Ejection fraction, *SSR* strain rate in systole, *E* early diastolic transmitral blood flow velocity, *A* late diastolic transmitral blood flow velocity, *E*/*A* ratio of early to late diastolic transmitral blood flow velocity, *E′* early diastolic myocardial tissue velocity, *P value* < 0.05,*d* Cohen’s standardised difference in means/effect sizes, *I*^*2*^ (%) tau squared, *CI* confidence interval

### LV systolic function

Meta-analysis outcomes for variables associated with LV systolic function are presented in Table 2. There was a significant reduction in EF of − 0.8 (− 1.2 to − 0.5) indicating a global decrease in systolic function following prolonged endurance exercise. This is a similar response to that observed by Middelton et al. (2006). As EF is heavily influenced by preload and afterload, recent research has focussed more on systolic tissue velocities and/or myocardial ε as representative measures of global or regional LV systolic function. The meta-analysis outcome for *S*′ indicated no difference pre- to post-exercise (9.5 ± 0.8–9.7 ± 0.9 cm/s, *P* = 0.9). Data from studies assessing myocardial systolic tissue velocities are equivocal with studies either reporting a reduction in *S*′ (Dawson et al. 2008; Scott et al. 2009; Chan-Dewar et al. 2010), no change (Oosthuyse et al. 2012) and even an increased*S*′ (Sahlen et al. 2009) following a bout of endurance exercise. *S*′ is partially mediated by HR and blood pressure, so study-to-study differences in these actors may go some way to explaining the disparate response reported for *S*′. It is also pertinent to note that *S*′ data are derived from only a small area of the basal septum and thus may not reflect global changes in function.

The meta-analysis identified a significant reduction in longitudinal *ε* of − 0.9 (− 1.0 to − 0.5) and systolic strain rate (SSR) of − 0.9 (− 1.3 to − 0.5). Fewer studies have investigated circumferential *ε* (*n* = 4) and radial *ε* (*n* = 4) after endurance exercise, therefore the meta-analysis outcomes suggesting a non-significant reduction post-exercise should be treated more cautiously (see Table 2). George et al. (2009) demonstrated changes in LV *ε* and SSR in all planes with a greater reduction post-exercise in radial and circumferential motion in their study of Comrades Marathon runners (c. 360 min running). Similar responses were noted for LV longitudinal and radial ε and SSR following a marathon (Oxborough et al. 2010b) and 100 mile ultramarathon (Oxborough et al. 2011; La Gerche et al. 2012).

The results of the meta-analysis demonstrated a reduction in twist of − 1.0 (− 1.6 to − 0.3) following prolonged endurance exercise. LV twist reflects the amount of energy stored in the myocardium during systolic contraction which is then subsequently released during diastole (Weiner et al. 2010). All five studies included in the meta-analysis reported a decline in LV twist following endurance exercise (Nottin et al. 2009; Oxborough et al. 2011; Vitiello et al. 2013a, b; Lord et al. 2016) that reflects reduced systolic contraction as well as reducing the elastic recoil during untwist in early diastole. This may go some may to explaining reduced early filling during diastole which will be discussed in the subsequent section.

### LV diastolic function

The meta-analysis identified a decrease in peak E flow velocity of − 1.0 (− 1.4 to − 0.6) following exercise (Table 2). The meta-analysis outcomes for *E, A* and the *E*/*A* ratio reflect both a reduction in *E* and a compensation in *A* velocities. This provides further support for altered diastolic filling post-prolonged endurance exercise reported in a previous meta-analysis (Middleton et al. 2006) and narrative review (Oxborough et al. 2010a, b). The decline in early LV filling and the compensatory increase in atrial contribution to LV filling may be mediated by a decline in preload and/or changes in intrinsic LV relaxation and compliance.

The meta-analysis also noted a reduction in *E*′ of − 0.7 (− 0.5 to − 1.0; Table 2) following exercise. A highly consistent reduction in*E*′ has been reported in response to prolonged endurance exercise (Nottin et al. 2009, 2012; Shave et al. 2009; Chan-Dewar et al. 2010; Vitiello et al. 2013a, b; Lord et al. 2016).

Given the small number of studies available early and late diastolic SR and LV untwist were not included in the meta-analysis. A decline in early diastolic SR has been reported alongside a reduction in LV untwist following a marathon (Oxborough et al. 2010b), ironman triathlon (Nottin et al. 2009) and 100 mile race (Oxborough et al. 2011).

### Possible mechanisms

The meta-analysis supports a growing evidence base that prolonged exercise can result in a significant and transient decrement in LV systolic and diastolic function and mechanics. Since the earliest human studies in this field there has been speculation, but very little empirical evidence, as to what combination of factors underpin this phenomenon (Dawson et al. 2003). The propensity for descriptive studies, largely in a field-based setting, has meant mechanistic insight has been limited given the significant limitations of field-based studies. To date, there has been suggestion that the following factors may have independent or synergistic roles to play; (1) post-exercise alterations in loading and heart rate, (2) subclinical levels of cardiomyocyte damage, (3) β-adrenergic desensitisation, and/or (4) serial or parallel ventricular interaction. We will briefly touch on all of these and detail the strength or lack of evidence related to these factors.

The role of altered rate or loading has been raised since the earliest empirical studies, as we are aware that prolonged exercise will likely lead to a raised HR, a relative hypotension and reduced LVEDV (preload) for a significant period post-exercise (Dawson et al. 2003). It is quite difficult to “unpick” the impact of changes in rate and loading from each other and alternative mechanisms that are occurring at the same time. Despite this there is evidence that point to the fact that changes in rate or loading cannot account for all of the changes in LV systolic and diastolic function or mechanics after endurance exercise. For example, the use of preload augmentation, via the Trendelenburg manoeuvre, after a marathon only normalised *E*/*A* and not *E*′ suggesting some degree of intrinsic impairment in relaxation (Hart et al. 2007). George et al. (2009) reported on a case series where ε was reduced to a greater extent in septal wall segments compared to the rest of the LV wall after a 90 km run. This localised impact on cardiac function suggested a localised intrinsic, rather than a global load-related mechanism. Chan-Dewar et al. (2010) reported an increase in electro-mechanical delay (the time between the electrical signal for contraction and peak tissue velocities in the LV wall) post-exercise that points to an intrinsic post-excitation mechanism. In addition, the approach often used in individual studies to correlate changes in LV function post-exercise with changes in rate and loading have been highly variable (Middleton et al. 2006). Whilst rate and loading may have some small role to play in mediating changes in LV function and mechanics after prolonged exercise, there is no strong evidence to suggest this is responsible for all of the changes observed. Consequently, researchers have looked for other mechanistic evidence, largely via indirect association.

Evidence of a role for myocardial damage/stunning (Shave et al. 2010; Scharhag et al. 2013) has largely involved descriptive data with the release/appearance of cardiac-specific biomarkers of myocyte damage (cardiac troponins; cTn) evident alongside changes in LV function and mechanics. Despite this, only two studies have demonstrated relationships between the degree of LV dysfunction and magnitude of troponin release following prolonged endurance exercise (Rifai et al. 1999; Neilan et al. 2006a, b). Most evidence does not support a direct temporal correlation of these two phenomena and therefore a causative effect of localised subclinical levels of cardiomyocyte damage on changes in function and mechanics is improbable and this is likely a benign response to elevated myocardial stress during prolonged endurance exercise (Shave et al. 2010).

Β-adrenergic receptor downregulation in response to sustained elevations in circulating catecholamines has been suggested as an alternative mechanism to explain LV dysfunction following prolonged endurance exercise. A number of studies have suggested that the increased circulating catecholamines during prolonged exercise may result in decreased cardiac β-receptor function or desensitisation via downregulation and/or uncoupling with the subsequent reduction in LV inotropic and chronotropic response explaining LV dysfunction (Eysmann et al. 1996; Douglas et al. 1998; Welsh et al. 2005; Hart et al. 2006). Whilst β-adrenergic receptor responsiveness cannot be directly assessed in athletes, previous studies have demonstrated a significant increase in the dose of both chronotropic and inotropic stimulant drugs required to generate the same change in heart rate or contractility pre- to post-prolonged endurance exercise inferring a reduction in cardiac β-receptor response following prolonged strenuous exercise (Eysmann et al. 1996; Douglas et al. 1998; Welsh et al. 2005; Scott et al. 2007). This downregulation has been related to systolic functional changes post-exercise (Welsh et al. 2005; Banks et al. 2010); however, it is not clear if any relationship exists with changes in diastolic function (Hart et al. 2006). As diastolic changes are prevalent following exercise of shorter duration than included in this meta-analysis (Hart et al. 2006), this would suggest that β-adrenergic downregulation may not fully explain changes in relaxation but may have a role in contractile change following longer duration exercise where receptors are exposed to high levels of circulating catecholamines over a longer time period.

More recently, a new theory related to LV and right ventricular (RV) interaction (serial or parallel) has emerged based on work from Oxborough et al. (2010b, 2011) and La Gerche et al. (2011, 2012). Data suggests a relatively higher elevation of pulmonary artery pressure (PAP) and therefore a disproportionately higher stroke work load in the RV compared to the LV during prolonged dynamic exercise (La Gerche et al. 2011). The thin walled RV myocardium may not able to sustain contractile force against an elevated afterload for a prolonged time period (La Gerche et al. 2011; Oxborough et al. 2011). This could lead to a reduction in RV contractility that would reduce the volume of blood that the RV is able to eject and, therefore downstream or “serially” LA preload will be impaired (Oxborough et al. 2010b). A drop in LA preload will lead to a reduced LV filling and subsequently impact upon LV systolic function and mechanics. Landmark studies by Oxborough et al. (2011) and La Gerche et al. (2012) reported RV dilatation and reduced RV*ε* with a concomitant reduction in LV EDV following prolonged exercise. There may also be a “parallel” component to ventricular interaction. In patients with a chronically elevated RV afterload the RV is dilated (Olson et al. 2010) and RV pressure may be elevated. The RV dilatation places an unbalanced volume overload on the interventricular septum and causes septal flattening or displacement, specifically in diastole (Ryan et al. 1985). A flattened interventricular septum may affect the structural integrity of the LV and impact on LV longitudinal and twist mechanics. This could also reduce the suction effect and pressure gradient caused by early LV relaxation and reduce LV filling. Evidence of septal displacement is reported by both La Gerche et al. (2012) and Oxborough et al. (2011) following prolonged endurance exercise, highlighting the likelihood of both a serial and parallel impact of the RV response to a relative elevation in pulmonary afterload.

## Limitations

This meta-analysis focused on LV mechanics and did not include a comprehensive review of global cardiovascular parameters (e.g., total peripheral resistance) whose reporting in ultra-endurance exercise studies is inconsistent. Future empirical work may address the interaction of cardiac mechanics within a holistic assessment of global cardiovascular function, as this may help unpick the implications (if any) of exercise-induced changes in cardiac mechanics.

## Conclusion

There is a growing body of evidence in support of a transient decline in LV systolic and diastolic function and mechanics following a period of ultra-endurance exercise. The current meta-analysis and narrative commentary add to this database with the key outcomes from the meta-analysis supporting a global reduction in LV systolic and diastolic mechanics following prolonged endurance exercise. Whilst the magnitude of this change is large enough to have a significant impact on function, this does not reach a level indicative of pathology; however, the clinical relevance of this diminished function after repeated bouts of endurance exercise is not fully understood. The mechanism(s) responsible for these changes are complex and likely multi-factorial in nature. Newer echocardiographic assessments may be able to provide some insight into these mechanism(s) in humans. Further studies are required in this complex and often contradictory field.

## Abbreviations

*A*′: Late diastolic myocardial tissue velocity
ASR: Late diastolic strain rate
CI: Confidence interval
cTn: Cardiac troponin
DBP: Diastolic blood pressure
*E*′: Early diastolic myocardial tissue velocity
*E*/*A*: Ratio of early to late diastolic transmitral blood flow velocities
EDV: End diastolic volume
EF: Ejection fraction
EICF: Exercise-induced cardiac fatigue
ESR: Early diastolic strain rate
Ɛ: Strain
HR: Heart rate
LV: Left ventricle
RV: Right ventricle
*S*′: Systolic myocardial tissue velocity
SBP: Systolic blood pressure
SR: Strain rate
SSR: Systolic strain rate
TDI: Tissue Doppler imaging
WMD: Weighted mean difference
